# Society Meetings

**Published:** 1907-01

**Authors:** 


### SOCIETY MEETINGS.

The German Hospital, of Buffalo, held the annual meeting of its directors November 30, 1906, when the following-named officers were chosen: president, William F. Kasting; vice-presidents, C. H. W. Auel and Joseph G. Schaff; secretary, Harry J. Knepper; treasurer, Henry C. Zeller; attorney, Jacob Stern; auditing committee, Ottomar Reinecke, Jacob J. Lang, and John Messersmith.

The directors are: Joseph Block, George Bleistein, Victor B. Blehdon, Edward C. Becker, John Brunner, Fred J. Dorn, Louis Fuhrmann, S. Gainsburg, William Germann, Herman E. Hayd, William Kreiner, Alex Kercher, John Kam, Frank Kraft, Jacob Gerhard Lang, Gerhard Lang, E. G. S. Miller, Louis W. Marcus, Anthony Miller, J. G. Meidenbauer, Charles Rohde, William T. Roberts, George Reimann, William Simon, Robert F. Schelling, Phillip Scheeler, C. A. Strangmann, Henry Steul, A. Schreiber, M. Schwartzmeier, William F. Wendt, Edward A. Weppner.

---

The Buffalo Academy of Medicine held meetings during the month of December, 1906, as follows:

*Section on Surgery.*—Tuesday, December 4, at 8:30 P. M. Program: (a) Somatic nerve manifestations of visceral disease, James A. Gibson; (b) Treatment of the stump in operations for appendicitis, Vertner Kenerson.

*Section on Medicine.*—Tuesday, December 11, at 8:30 P. M. Program: (a) The acute infectious paralyses, James W. Putnam; (b) Medical ethics within and without the profession, John D. Bonnar.

*Section on Obstetrics and Gynecology.*—Tuesday, December 18, at 8:30 P. M. Program: (a) Reflex dyspepsia due to abdominal and pelvic diseases, Herman E. Hayd; (b) Melena neonatorum, William G. Taylor.

---

The Medical Society of the State of New York will hold its one hundred and first annual meeting at Albany, Tuesday, Wednesday and Thursday, January 29, 30 and 31, 1907, under the presidency of Dr. Joseph D. Bryant, of New York. Dr. Wisner R. Townsend, 64 Madison Avenue, New York, is the secretary. Dr. L. H. Neuman, 194 State Street, Albany, is the chairman of the committee on scientific work, who should be addressed on matters pertaining thereto.

---

The Southern Surgical and Gynecological Association, at its recent annual meeting, held at Baltimore, elected the following-named officers: president, Howard A. Kelly, Baltimore; vice-president, R. E. Fort, Nashville; secretary, William D. Haggard, Nashville; treasurer, Charles M. Rosser, Dallas, Texas. The next annual meeting will be held at New Orleans, in December, 1907.

The Medical Society of the County of Erie will hold its eighty-sixth annual meeting on Tuesday, January 8, 1907, at the Buffalo public library building at 10 o'clock A. M. The president is Dr. A. H. Briggs and the secretary is Dr. F. C. Gram. At this writing, December 24, no announcement nor program has been issued.

